# Contribution of the Mitochondrial Carbonic Anhydrase (MoCA1) to Conidiogenesis and Pathogenesis in *Magnaporthe oryzae*

**DOI:** 10.3389/fmicb.2022.845570

**Published:** 2022-02-17

**Authors:** Yuejia Dang, Yi Wei, Wajjiha Batool, Xicen Sun, Xiaoqian Li, Shi-Hong Zhang

**Affiliations:** ^1^Center for Extreme-Environmental Microorganisms, Shenyang Agricultural University, Shenyang, China; ^2^College of Plant Protection, Shenyang Agricultural University, Shenyang, China

**Keywords:** carbonic anhydrase, mitochondrion, conidiophore, pathogenesis, *Magnaporthe oryzae*

## Abstract

The interconversion of CO_2_ and HCO_3_^−^ catalyzed by carbonic anhydrases (CAs) is a fundamental biochemical process in organisms. During mammalian–pathogen interaction, both host and pathogen CAs play vital roles in resistance and pathogenesis; during planta–pathogen interaction, however, plant CAs function in host resistance but whether pathogen CAs are involved in pathogenesis is unknown. Here, we biologically characterized the *Magnaporthe oryzae* CA (MoCA1). Through detecting the DsRED-tagged proteins, we observed the fusion MoCA1 in the mitochondria of *M. oryzae*. Together with the measurement of CA activity, we confirmed that MoCA1 is a mitochondrial zinc-binding *CA*. *MoCA1* expression, upregulated with H_2_O_2_ or NaHCO_3_ treatment, also showed a drastic upregulation during conidiogenesis and pathogenesis. When *MoCA1* was deleted, the mutant Δ*MoCA1* was defective in conidiophore development and pathogenicity. 3,3′-Diaminobenzidine (DAB) staining indicated that more H_2_O_2_ accumulated in Δ*MoCA1*; accordingly, *ATPase* genes were downregulated and ATP content decreased in Δ*MoCA1*. Summarily, our data proved the involvement of the mitochondrial *MoCA1* in conidiogenesis and pathogenesis in the rice blast fungus. Considering the previously reported HCO_3_^−^ transporter MoAE4, we propose that *MoCA1* in cooperation with *MoAE4* constitutes a HCO_3_^−^ homeostasis-mediated disease pathway, in which *MoCA1* and *MoAE4* can be a drug target for disease control.

## Introduction

Carbon dioxide (CO_2_), a fundamental physiological gas for all the living organisms, is a vital component of carbon cycle. It is not only a waste product of cellular respiration but also a nutrient regulator and a stimulant molecule in different signaling pathways (Jones, 2008). In living organisms, the concentration of CO_2_ is balanced by a highly diverse class of enzymes known as carbonic anhydrases. The carbonic anhydrases termed CAs belong to the metalloenzymes that catalyze the interconversion of carbon dioxide hydration and bicarbonate ions (CO_2_ + H_2_O ↔ HCO_3_^−^ + H^+^; Elleuche and Pöggeler, 2009), by which the interconversion reaction can be accelerated at a high rate up to 10,000-fold to ensure adequate level of CO_2_ or HCO_3_^−^ as the substrates for other enzymatic reactions (Wistrand, 1981; Cronk et al., 2001).

In terms of the complex classification of the CA enzymes, CA belongs to a large protein family. Members of CA family are structurally unrelated enzymes, sharing low sequence similarity but possessing a quite similar active sites-based architecture (Dostál et al., 2018). To date, the CA family has been divided into eight evolutionary independent classes (α, β, γ, δ, ζ, η, θ, and ι; Dreyer et al., 2020). As a matter of fact, according to the amino acid composition of the metal coordination sphere, all CAs can be simply divided into α-like and β-like CAs (Polishchuk, 2021). The α-like CAs includes α-, γ-, δ-, η-CAs; and the β-like CAs includes β-, ζ-, θ-CAs (Polishchuk, 2021). Base on the studied CAs, the α-class CAs were found in prokaryotes, protozoa, fungi, plants, and mammals (Dostál et al., 2018; Supuran and Capasso, 2020); the β-class CAs were found in all other types of organisms except for mammals (Elleuche and Pöggeler, 2010; Supuran, 2018a; Dreyer et al., 2020). These enzymes display a catalytic zinc ion coordinated by the three highly conserved residues two Cys and one His (Kimber, 2000; Teng et al., 2009) and are mostly localized to the cytosol, plasma membrane, and mitochondria whereas some isoforms in the chloroplast (Dostál et al., 2020). Sometimes, the β-class CAs can be further divided into the plant-like β-CAs, the cab-like β-CAs, and the ε-class β-CAs, all of which are from prokaryotes (Kimber, 2000; Sawaya et al., 2006).

Various isoforms of CAs, reported in different organisms, are involved in a series of fundamental biological processes, pH homeostasis, and even bio-/abio-stresses (Teng et al., 2009; Elleuche and Pöggeler, 2010; Supuran, 2018a; Polishchuk, 2021). In mammals, CAs play critical roles in oxygen transport, pH regulation, and ion exchange (Oosterwijk, 2014). In plants, CAs are involved mainly in photosynthesis and respiration, as well as in stress-related changes, such as drought, high salinity, heat, light, excess bicarbonate, and pathogen responses (Polishchuk, 2021). Thus, CA expression responds to environmental stresses and is related to stress tolerance in plants (Yu et al., 2007). The present findings support the hypothesis that CAs function to facilitate the diffusion of CO_2_ to the site of inorganic carbon fixation in rice plant (Sasaki et al., 1998; Floryszak-Wieczorek and Arasimowicz-Jelonek, 2017). And in fungal pathogens, CAs generally participate in the CO_2_-sensing system of fungus and in the regulation of sexual development (Elleuche and Pöggeler, 2010).

The in-depth study of fungal CAs is only in recent years. Both α and β-CAs have been found in fungi (Lehneck and Pöggeler, 2014). Fungal β-CAs play an essential role in growth, differentiation, survival, and virulence by catalyzing the reversible mutual conversion of CO_2_ and HCO_3_^−^ (Kim et al., 2020). In hemiascomycetous yeasts, the *Nce103* gene encoding a plant-type β-CA is required for fungal growth specifically under CO_2_ condition but is not essential for pH homeostasis at high CO_2_ levels (Klengel et al., 2005; Innocenti et al., 2009). In addition to the model fungi yeast, β-class CAs from human pathogenic fungi have been intensively studied. *Candida albicans*, the β-CA works as a CO_2_ scavenger essential for pathogenicity in niches where the available CO_2_ is limited, such as epithelial cell surfaces (Klengel et al., 2005). Studies on fungal β-CAs, CAN1 and CAN2 of *Cryptococcus neoformans* and *Cryptococcus gattii*, showed that the two fungal pathogens are involved in CO_2_ sensing and virulence to human hosts (Bahn et al., 2005; Han et al., 2010; Ren et al., 2014). Similarly, in the human pathogenic filamentous fungi *Aspergillus fumigatus* and *Aspergillus nidulans*, CAs disruption could also affect *Aspergilli* conidiation and virulent infection (Han et al., 2010). Interestingly, in a filamentous fungal model *Sordaria macrospora*, the four identified β-CAs (CAS1-4) have been characterized, which demonstrated to be related to the vegetative growth, ascospore germination, and sexual development (Elleuche and Pöggeler, 2009). Therefore, fungal CAs are crucial not only for cell survival and proliferation, but also for various CO_2_-related signaling cascades that are important for virulence and differentiation of pathogenic fungi (Han et al., 2010).

Plant β-CAs have been implicated in plant CO_2_ metabolism, development, and host resistance (Zhou et al., 2020). When potato CA-silenced lines were inoculated by using the oomycete *Phytophthora infestans*, potato late blight disease occurred seriously with the rapid growth and reproduction of *P. infestans*, indicating that suppression of CA increases susceptibility to the pathogen (Restrepo et al., 2005). During planta–pathogen interactions, plant CAs function in host resistance but whether pathogen CAs are involved in pathogenesis is unknown. The pathogen fungus usually undergoes host physiological adversity resistance, such as nitrogen starvation, high HCO_3_^−^, and low-oxygen stress during its invasive hyphal growth and infectious development in host plant (Hammond-Kosack and Parker, 2003; Egan et al., 2007). To colonize the host successfully, pathogen fungus must ensure a basic strategy to survive these adverse environmental conditions and sensing CO_2_ level is one of those strategies. Till now, plant pathogenic fungal CAs have not performed in plant–fungal pathogen system, though the relationship between plant CAs and host resistance is being revealed. In our previous study, we delineated a cytomembrane and tonoplast located HCO_3_^−^ transporter MoAE4, which is required for development and pathogenicity in *Magnaporthe oryzae* (Dang et al., 2021b). Based on the MoAE4 research, we assumed that *M. oryzae* β-CA (MoCA1) gene is involved in pathogenesis of the blast fungus. In this study, we functionally biologically and genetically characterize the MoCA1 in *M. oryzae*. The relationships between *MoAE4* and *MoCA1* in the blast fungus development and pathogenesis are also discussed.

## Materials and Methods

### Sequence Alignment Assays

The *MoCA1* (MGG_04611) gene and amino acid sequences were acquired from the NCBI database.^1^ The protein tertiary and subcellular location prediction were predicted using I-TASSER^2^ and Softberry.^3^ In addition, the amino acid sequence was aligned using the DNAMAN program, and the phylogenic tree was drawn using MEGA7.0.9 software.

### Fungal Strains and Culture Conditions

*Magnaporthe oryzae* strain JJ88 was used as wild type and was isolated and purified from *Oryza sativa* cultivar Jijing88, a variety that is widely planted in Jilin Province, China. All the fungal strains were cultured on complete media (CM) agar plates and kept on filter papers at −20°C {CM [10 g/L glucose, 2 g/L peptone, 1 g/L yeast extract, 1 g/L casamino acids, 0.1% (V/V) trace elements, 0.1% (V/V) vitamin supplement, 0.5 g/L MgSO_4_, 6 g/L NaNO_3_, 0.5 g/L KCl, and 1.5 g/L KH_2_PO_4_, pH 6.5]}. For conidiation, the strains were inoculated on oatmeal–tomato agar medium (OMA) at 24°C for 7 days in the dark (Dang et al., 2021a). The strains were grown continually for 3 days while illuminated under fluorescent lights after the aerial hyphae of the strains had been removed by washing with sterile distilled water.

### Prokaryotic Expression and Enzyme Activity Determination

For recombinant protein preparation, the full-length *MoCA1* cDNA was amplified by PCR using a pair of primers (MoCA1-C-S/A), comprising *BamH* I and *Sal* I restriction sites. The PCR product was subcloned into the pMD-19T vector (TaKaRa, Dalian, China). The *BamH*I and *Sal*I fragment of pMD-19T, possessing the open reading frame of *MoCA1*, was cloned into the pET-28a (+) vector (Novagen, Shanghai, China). *Escherichia coli* cells harboring the pET-28a:: MoCA1 plasmid were transferred in BL21 (DE3) and grown in the LB medium at 37°C. Once the cell density at OD600 reached to 0.6, IPTG was added to a final concentration of 1 mM and continued to culture for another 3–4 h. Recombinant protein was purified using a Ni^2+^-NTA purification kit according to the product instructions (Novagen, Shanghai, China).

The purified native MoCA1 was normalized to a concentration of 1 mg/ml. For this assay, Tris buffer and BSA were used as a blank and negative control, respectively, carbonic anhydrase from bovine erythrocytes (BCA; Merck, Shanghai, China) was used as a positive control. The improve method from Wilbur and Anderson was used (Sridharan et al., 2021) that involved monitoring of time taken during the pH change of 12 mM Tris Buffer at 0°C from 8.3 to 6.3 in the presence of carbonic anhydrase. CO_2_ bubbled double distilled water was used as the substrate for this reaction. The experiment was performed with 6 ml of chilled buffer, 4 ml of CO_2,_ and 100 μl of CA enzyme added different 10 mM metal ion as catalytic agents. The activity was calculated using the following formula, Activity in WAU = 2*(T_0_−T)/T* mg of Enzyme. Where T_0_ is the duration taken by Blank (Buffer).

### Assays for the Subcellular Localization of *MoCA1*

The localization of *MoCA1* was observed by tagging it with the *BamH* I-*Sma* I sites of red fluorescent protein (RFP) of vector pKD7-Red. Later, we generated transgenic strains expressing RFP-tagged *MoCA1* fusion gene in the knockout mutant of *M. oryzae* (pKD7-MoCA1:: RFP). Fluorescent microscopic observation was carried out by using hyphae (6 days) and conidia (6 days). To visualize the mitochondria, vegetative hyphae, and conidia were treated with 1 mM Mito-Tracker Green (Beyotime, Shanghai, China) solution for 15–45 min at 37°C before observed under laser scanning confocal microscope (Olympus fluoview FV3000, Olympus, Tokyo, Japan).

### Targeted Gene Deletion and Complementation

To generate the *MoCA1* replacement construct pXEH2.0, the upstream (1,220 bp) and downstream (1,445 bp) fragments of *MoCA1* were amplified using primers MoCA1-L-S/MoCA1-L-A and MoCA1-R-S/MoCA1-R-A, respectively. The resulting PCR products were cloned into the *Bgl* II-*EcoR* I and *Xba* I-*Pst* I sites of vector pXEH2.0. The knockout vector was introduced into *Agrobacterium tumefaciens* strain AGL-1 and then transformed into the wild-type *M. oryzae* using the *A. tumefaciens*-mediated transformation (ATMT) method as previously described (Dang et al., 2021b). Transformants were selected and cultured in 200 μg/ml hygromycin. The transformants were identified using PCR with primers HYG-S/HYG-A, MoCA1-LHYG-S/MoCA1-LHYG-A, and MoCA1-G-S/MoCA1-G-A.

The entire *MoCA1* sequence was amplified using a PCR technique with MoCA1-C-S/MoCA1-C-A and inserted into the hygromycin resistant vector pKD7 for complementation into the mutant strain. The reconstructed pKD7-*MoCA1* was transformed into the Δ*MoCA1* mutant strain and designated Δ*MoCA1*/*MoCA1*. The complemented strain was confirmed by PCR with MoCA1-G-S/MoCA1-G-A.

To further verify the gene deletion and complementation, the expression of the wild-type, Δ*MoCA1* mutant, and Δ*MoCA1*/*MoCA1* strains was amplified using qRT-PCR with qRT-MoCA1-S/qRT-MoCA1-A and Actin-S/Actin-A, and the strains were identified. The primers for gene deletion and complementation are listed in Supplementary Table S2.

### Quantitative Real-Time PCR

The total RNA was isolated from mycelia that had been harvested from 5-day-old CM media using the TRIzol reagent (Invitrogen, Carlsbad, CA, United States). First-strand cDNA was synthesized using an oligo (dT) primer from total RNA, which had been treated with DNase I. Subsequently, qRT-PCR was performed using an ABI7500 System (Applied Biosystems, Foster City, CA, United States) and SYBR Premix Ex Taq (TaKaRa, Dalian, China). The relative mRNA levels were calculated using the 2^−ΔΔCq^ (C_q_ = C_qgene_−C_qactin_) method. The *M. oryzae* actin gene (MGG_03982.6) was utilized as a reference gene for normalization. Each sample was tested in three replicates in each experiment. The primer sequences used for qRT-PCR are shown in Supplementary Table S2.

### Assays for Conidial Production, Growth, and Development

The strains (wild type, Δ*MoCA1*, and Δ*MoCA1*/*MoCA1*) were cultured on OMA media as previously described (Dang et al., 2021b). After 3 days of cultivation at 28°C, sterile water was added to remove the hyphae, and a piece of the culture medium was cut with a blade and placed on a glass slide. It was then placed in a moisturizing box and incubated at 28°C. The prepared sample was then observed under a Nikon Eclipse 80i microscope at 12, 24, 48, and 72 h. The strains were then stained with lactophenol cotton blue to observe the conidiophore stalks and hyphae under a light microscope (Dang et al., 2021b). Additionally, the conidia were collected with 2 ml of sterile water after 3 days of culture on OMA media and counted with a hemocytometer. Each strain was repeated three times, and the experiment was conducted in triplicate.

Conidia of the wild type, Δ*MoCA1*, and Δ*MoCA1*/*MoCA1* were cultured on OMA media and collected to observe the germination of conidia and formation of appressoria. The conidial suspension was adjusted to 1 × 10^5^/ml and added drop wise to a hydrophobic cover slip under a microscope at 1, 2, 3, 4, and 6 h. Three biological replicates of each strain were used, and the experiment was conducted in triplicate.

### Rice Sheath Penetration and Plant Infection Assays

To determine the pathogenicity of *MoCA1*, the wild-type, Δ*MoCA1*, and Δ*MoCA1*/*MoCA1* strains were inoculated on OMA media to collect the conidia as previously described. The fourth leaf stage of rice seedlings (*O. sativa* cv. Lijiangxintuanheigu) was assayed for infection following, spraying of 2 ml of a conidial suspension (5 × 10^4^ conidia/ml in 0.2% gelatin). The inoculated plants were placed in the dark in a dew chamber for 24 h at 28°C and then transferred to a growth chamber with a photoperiod of 16 h for 7 days post-inoculation (dpi).

Conidial suspensions (100 μl, 5 × 10^4^ conidia/ml) were injected into seedling leaf sheaths, and the inoculated plants were placed in a moist chamber as described previously. The formation of lesions and necrosis around the inoculation sites was examined when the injection-wounded leaves unfolded at different time points after the injection. The mean infectious hyphae (IH) growth rates and movement to the adjacent cells were determined from 100 germinated conidia per treatment at 12, 24, and 48 h post-inoculation (hpi) and repeated in triplicate as previously described. The leaf sheaths were trimmed at the time points indicated and observed using a Nikon Eclipse 80i microscope. This experiment was performed with three independent replicates, and the representative results from one of these experiments are presented.

### Assays for H_2_O_2_ and NaHCO_3_ Treatment

To illustrate the effect of different concentrations of H_2_O_2_ and NaHCO_3_ on the expression of *MoCA1* gene, the concentrations were used in previous study (Dang et al., 2021b), wild-type strains of *M. oryzae* were cultivated on CM agar that contained 2.5, 5, and 7.5 mM H_2_O_2_, and on PDA with 0, 12.5, 25, 37.5, 50, 62.5, and 75 mM NaHCO_3_ at 28°C for 7 days. And the wild type of mycelium treated under different concentrations of H_2_O_2_ and NaHCO_3_ was collected for expression patterns of MoCA1.

### Extraction and Purification of Melanin

The method was used for extracting the pigment from the conidia and appressoria. Collect the conidia and appressoria (1 × 10^6^/ml) and centrifuge (5,000 rpm, 5 min). After the supernatant is removed, the precipitate was dried and weighed 0.05 g of the precipitate was added to 6 ml of 1 M NaOH solution according to 1:120 (w/v). Continue heating at 121°C for 20 min to extract melanin. With 1 M NaOH as a blank control, the absorbance was measured at 405 nm with an ultraviolet spectrophotometer (Implen N50, Germany; Suryanarayanan et al., 2004).

### H_2_O_2_ Treatment and Endogenous H_2_O_2_ Measurements

The H_2_O_2_ content was determined as previously described (Dang et al., 2021b). H_2_O_2_ was extracted by homogenizing 3 g of mycelia from the wild-type, Δ*MoCA1*, and Δ*MoCA1*/*MoCA1* strains in 6 ml of cold acetone. The homogenate was then centrifuged at 3,500 rpm for 5 min at room temperature, and the resulting supernatant was designated as the sample extract. Next, 0.1 ml of titanium reagent [5% (w/v) titanic sulfate in concentrated H_2_SO_4_] was added to 1 ml of the sample extract, followed by the addition of 0.2 ml of strong aqueous ammonia to precipitate the peroxide–titanium complex. The precipitated sample was centrifuged at 3,000 rpm for 10 min at room temperature; the supernatant was discarded, and the precipitate was then solubilized in 5 ml of 2 M H_2_SO_4_. The absorbance of the samples was determined at 415 nm against a blank of 2 M H_2_SO_4_. The H_2_O_2_ concentration in the samples was determined by comparing the absorbance against a standard curve of a 0–5 mM titanium–H_2_O_2_ complex that was prepared according to Cui et al. (2017).

The production of H_2_O_2_ was monitored by staining with 3,3′-diaminobenzidine (DAB) as previously described (Dang et al., 2021b). The hyphae of the wild-type, Δ*MoCA1*, and Δ*MoCA1*/*MoCA1* strains were cultured in CM media for 5 days and then incubated in the dark in a 1 mg/ml solution of DAB at room temperature for 8 h. The samples were washed with sterile water and observed under a Nikon light microscope. This experiment was performed in triplicate and repeated three times for each strain. Similarly, leaf sheath cells of rice infected by wild-type, mutant, and complementation strains were stained DAB at 36 hpi.

### Determination of Intracellular ATP at Different Stages of *Magnaporthe oryzae*

The production of ATP was monitored using an ATP Bioluminescence Assay Kit (Beyotime, Shanghai, China) to define the diversification of content of ATP in mycelia and conidia of *M. oryzae*.

### Statistical Analysis

All the experiments were performed at least three times. The mean ± SD of the strain diameter, germination rate, and relative expression were determined using SPSS Statistics 22 (IBM, Inc., Armonk, NY, United States). Error bars represent the SD. ns indicates no significant difference at *p* > 0.05. *indicates a statistically significant difference at *p* < 0.05. **indicates a highly significant difference at *p* < 0.01. ***indicates a highly significant difference at *p* < 0.001.

## Results

### MGG_04611 (MoCA1) Encodes a Zinc-Activated Carbonic Anhydrase in *Magnaporthe oryzae*

Homologous sequences of β-CA proteins have been reported in a variety of species. Based on the conserved amino acid sequences of several reported β-CA proteins, a putative zinc-activated carbonic anhydrase protein β-CA (MGG_04611) was searched in the *M. oryzae* genome.^4^ The *M. oryzae* β-CA, termed as MoCA1, with a length of 2,594 bp open reading frame, encodes a protein of 232 amino acids.

**Figure 1:**
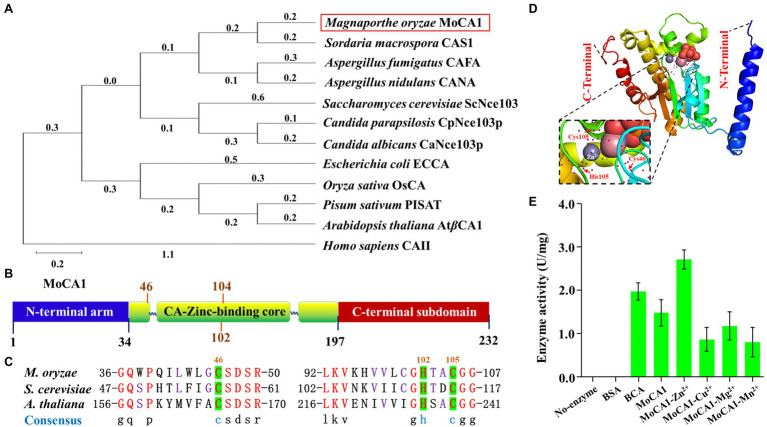
Structure of the MoCA1, phylogenetic analysis, and identification of MoCA1. **(A)** Phylogenetic tree constructed with β-CA proteins homologs from *Magnaporthe oryzae*, *Sordaria macrospora*, *Aspergillus fumigatus*, *Aspergillus nidulans*, *Candida parapsilosis*, *Candida albicans*, *Saccharomyces cerevisiae*, *Escherichia coli*, *Pisum sativum*, *Arabidopsis thaliana*, and *Homo sapiens*. **(B)** Structure of the MoCA1. MoCA1 is a Zn metalloenzyme. **(C)** Sequence alignment. A Zn ion coordinated by the three highly conserved residues two Cys and one His in *M. oryzae*, *S. cerevisiae*, and *A. thaliana*. **(D)** Tertiary structure (3-D) of MoCA1. **(E)** Measurement of MoCA1 activity. Tris buffer was used as a blank, BSA was used as a negative control, BCA was used as a positive control, and each ion with MoCA1 was compared with each other.

Additional results from phylogenetic analysis indicated that MoCA1 was closely related to the fungal group (Figure 1A), sharing 67.23% identity with *S. macrospora* CAS1 gene. The MoCA1 is composed of three sequential components: an N-terminal arm, a conserved zinc-binding core (34–197), and a C-terminal subdomain (Figure 1B). The tertiary (3D) structures of MoCA1 and a zinc ion are coordinated by the three highly conserved amino acid residues Cys46, His102, and Cys104 as predicted with the web-based I-TASSER^5^ (Figures 1C,D).

As reported previously, β-CA is a Zn metalloenzyme that catalyzes the interconversion of carbon dioxide and water into bicarbonate and hydrogen ions with exceptionally high efficiency (Fabre et al., 2007; Dreyer et al., 2020). To confirm MoCA1 encodes a zinc-activated carbonic anhydrase, recombinant proteins of MoCA1 were expressed in *E. coli* and purified to homogeneity with a single-step process using a Ni^2+^-NTA (Supplementary Figure S1A). Enzymatic rates of CO_2_ hydration reaction catalyzed by recombinant β-CA enzyme with different metal ions, that is, Zn^2+^, Cu^2+^, Mg^2+^, Mn^2+^, were measured, the result indicated that MoCA1 activity with Zn^2+^ was significantly higher as compared to other metal ions (Figure 1E), which suggests that MoCA1 encodes a zinc-activated carbonic anhydrase and catalyzes CO_2_ hydration reaction efficiently.

### MoCA1 Subcellular Localization and Expression Patterns Under Different Conditions

**Figure 2:**
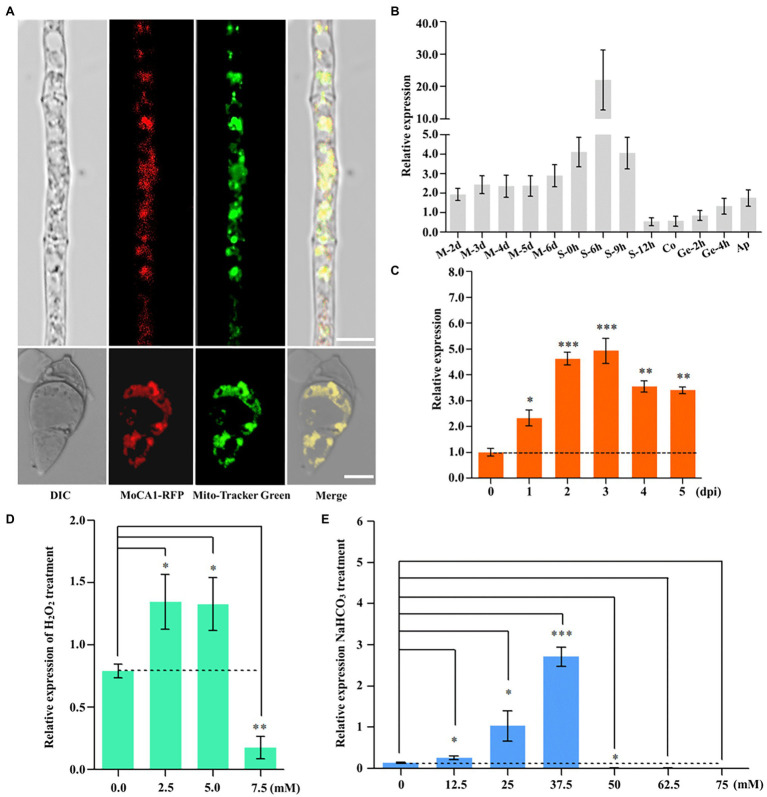
MoAE4 subcellular localization and function in *M. oryzae*. **(A)** Subcellular localization in hyphae and conidia (6 days). Red fluorescence signals of the MoCA1-RFP protein of 6-day-old hyphae and conidia were examined by confocal microscopy and co-localized with Mito-Tracker Green on mitochondria. Scale bar = 10 μm. **(B)** The expression pattern of growth of *M. oryzae*. M 0–6 days, mycelia 2–6 days; S 0–12 h, conidiophores 0–12 h; Co, conidia; Ge 2–4 h, germination 2–4 h; Ap, appressoria. **(C)** The expression pattern of infection development of *M. oryzae*. dpi, days post-inoculation. **(D)** Transcription abundance of MoCA1 under different concentrations of H_2_O_2_. **(E)** Transcription abundance of MoCA1 under different concentrations of NaHCO_3_. Data represent the means ± SD from three independent experiments in which triplicate plates were examined for each strain in each experiment. ^*^p < 0.05; ^**^p < 0.01; and ^***^p < 0.001.

Previous studies have shown that β-CAs locate to the cytosol, plasma membrane, or mitochondrion (Dostál et al., 2020). Therefore, to confirm the subcellular localization of MoCA1, we generated transgenic strains expressing RFP-tagged MoCA1 fusion gene in the wild type of *M. oryzae* (Supplementary Figure S1B). Mito-Tracker Green (Beyotime, Shanghai, China) was used as a mitochondrial tracker. A strong red fluorescence signal of the MoCA1-RFP protein co-localized with green fluorescence of Mito-Tracker in fungal hyphae and conidia (Figure 2A) which indicates that MoCA1 localizes to mitochondria of *M. oryzae*.

Furthermore, to characterize the role of MoCA1 in fungal growth and pathogenic development, we assess the expression patterns of MoCA1 at different stages of *M. oryzae* life cycle, that is, during mycelia 2–6 days, conidiophore 0–9 h, conidium, germination 2–4 h, appressorium, and 0–7 days of infection cycle through qPCR. We found that the relative expression was significantly increased at conidiophore stage (Figure 2B) and was gradually increased during the first 3 days of the infection (Figure 2C). Collectively, these results reflect that MoCA1 is involved in the development of conidiophore stalks and plays its role during early stage of infection.

### MoCA1 Deletion Causes Defects in Conidiogenesis and Appressorium Development

**Figure 3:**
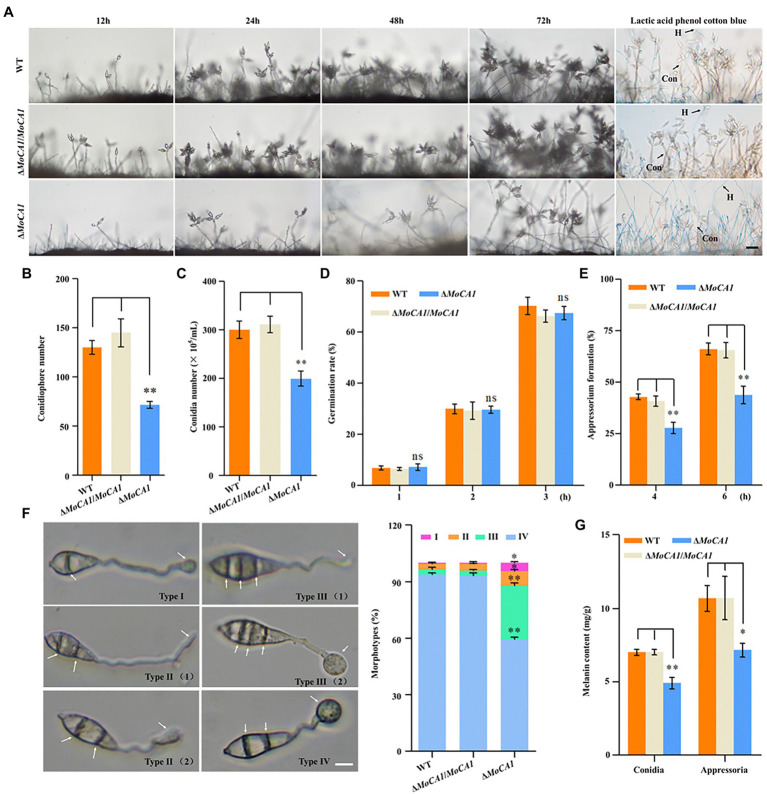
Conidia and appressoria development analysis of the wild-type and mutant strains. **(A)** Conidiophore stalks stained with lactophenol cotton blue. The conidiophores of the wild type, the Δ*MoCA1*, and Δ*MoCA1*/*MoCA1* strains induced for 12, 24, 48, and 72 h; strains were stained with lactophenol cotton blue at 72 h. The hyphae are stained blue, whereas the conidiophore stalks are in gray. Bar = 50 μm. **(B)** Statistical analysis of the conidiophores number of the wild type, the Δ*MoCA1*, and Δ*MoCA1*/*MoCA1* mutant strains. **(C)** Statistical analysis of conidial production in the strains. **(D)** Conidial germination rate. Conidial germination was calculated under the microscope at 1, 2, and 3 h. **(E)** Appressorial formation rate. Appressorial formation was calculated under the microscope at 4 and 6 h. **(F)** The statistics of appressorium morphological types. Scale bar = 10 μm. **(G)** The melanin content of strains in conidia and appressoria. The analysis was performed using an independent samples *t*-test. ^ns^*p* > 0.05; ^*^p < 0.05 and ^**^p < 0.01. Error bars indicate the mean ± SD from three independent experiments.

To ascertain the role of MoCA1 in physiological and pathological development of *M. oryzae* we generated the MoCA1 Knockout strains using the *A. tumefaciens*-mediated transformation (ATMT) method (Supplementary Figures S1C–F). Later, conidiation and appressorial formation were analyzed among the Δ*MoCA1*, Δ*MoCA1*/*MoCA1*, and wild-type strains. The sparse conidiophores with less number of conidia were observed in the *MoCA1* deleted strain as compared to wild type with almost 40%–60% the total number of conidiophores and conidia as of wild-type and Δ*MoCA1*/*MoCA1* strains (Figures 3A–C). The conidial germination rate of all strains including the wild type was similar at 1–3 h, (Figure 3D; Supplementary Figure S2B). In terms of appressorial formation, Δ*MoCA1* had a lower formation rate than that of wild type and Δ*MoCA1*/*MoCA1* (Figure 3E). As the results, MoCA1 is proposed to be involved in the conidiogenesis and appressorial formation.

As appressorium of *M. oryzae* is the key factor in infecting the host plant (Talbot, 2003), we observed the appressorium morphology to analyze effect of MoCA1 deletion on appressorium morphogenesis, our results showed that Δ*MoCA1* mutant displayed incomplete appressorium maturation. Four appressorium morphologies, type I (a septum in the conidium and appressorium formation), type II (two septa in the conidium and appressorium formation or immature), type III (three septa in the conidium and appressorium formation or immature), and type IV (normal morphology) were observed (Figure 3F). However, about 90% wild-type and Δ*MoCA1*/*MoCA1* appressoria were normal and only about 50% in Δ*MoCA1* mutant (Figure 3F). More importantly, the melanin of the conidia and appressoria of the three strains were extracted. Our data demonstrated that the melanin content of Δ*MoCA1* appressoria was 30% less compared to wild type and Δ*MoCA1*/*MoCA1* (Figure 3G). However, the melanization of mutant was not altered when observed at hyphae stage (Supplementary Figure S2A). Therefore, we infer that MoCA1 is involved in appressorium maturation and morphogenesis.

### MoCA1 Is Important for Pathogenicity in *Magnaporthe oryzae*

**Figure 4:**
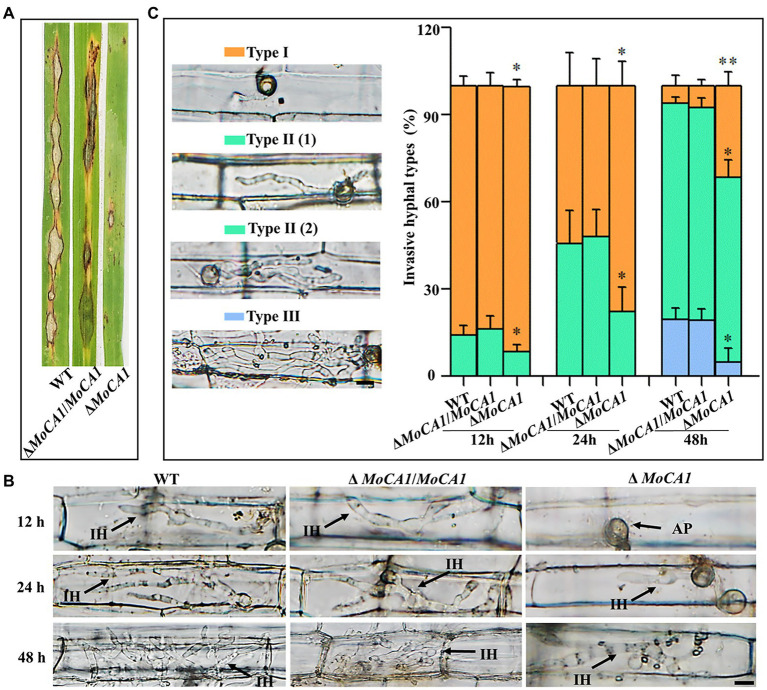
Pathogenesis analysis of the wild-type and created strains. **(A)** Spray inoculation assay. **(B)** Rice leaf sheath infection assay. The conidial suspension of indicated strains was injected into a rice sheath. Representative photographs of infectious hyphae were taken after 12, 24, and 48 h of incubation at 25°C. Scale bar = 10 μm. IH, infectious hyphae and AP, appressoria. **(C)** The infection rate was calculated according to the number of type I to type III events. The infection status of more than 100 germinated conidia per leaf sheath was scored at 12, 24, and 48 hpi. Type I, conidia with mature appressoria; Type II, primary hyphae formed, infectious hyphae extended and branched in one cell; Type III, infectious hyphae crossing to neighboring cells. Values represent the averages of five measurements ± SD. The statistical analysis was performed using a one-way ANOVA with Tukey’s multiple comparison test. The averages were taken from the quadruplicate analysis. Values are based on three biological samples, and error bars indicate SD, ^*^p < 0.05 and ^**^p < 0.01.

In order to identify the influence of appressorium maturation and morphology defects on pathogenic development of MoCA1 mutant, pathogenicity assays were carried out using conidia collected from Δ*MoCA1*, Δ*MoCA1*/*MoCA1*, and the wild-type strains. When intact susceptible rice seedlings were inoculated with conidial suspension, at 7 dpi, some acute expansive disease lesions were observed on rice leaves inoculated with the wild type and Δ*MoCA1*/*MoCA1*; however, Δ*MoCA1* inoculated rice leaves showed very few disease lesions as shown in Figure 4A.

Further, leaf sheath infection assays were performed to examine the infectious development of the MoCA1 deleted strains in host plant, rice (Figure 4B). To decipher the exact action of MoCA1 during pathogenic development, we defined the three types of infection hyphae according to their developmental morphologies (Figure 4B). Later we quantified the proportion of the three types of infection hyphae based on 100 germinated conidia in the inoculated leaf sheaths (Figure 4C). At 12 hpi, we observed that 15% of wild-type and Δ*MoCA1*/*MoCA1* spores formed invasive and primary infectious hyphae whereas less than 10% of Δ*MoCA1* spores were able to form primary infectious hyphae. At 24 hpi, about 45% of invasive and primary infectious hyphae were observed in wild-type and Δ*MoCA1*/*MoCA1* stains, whereas Δ*MoCA1* strains only had 15% invasive and primary infectious hyphae. Yet, at 48 hpi, Δ*MoCA1* strain had less invasive hyphae (types II and III) extended to neighboring cells compared to wild-type and Δ*MoCA1*/*MoCA1* strains (Figure 4B), which shows that the leaf penetration capabilities of *MoCA1* deleted strain was compromised. These results suggest the importance of MoCA1 in pathogenesis.

### MoCA1 Is Associated With Endogenous Hydrogen Peroxide Suppression and the ATP Supply

**Figure 5:**
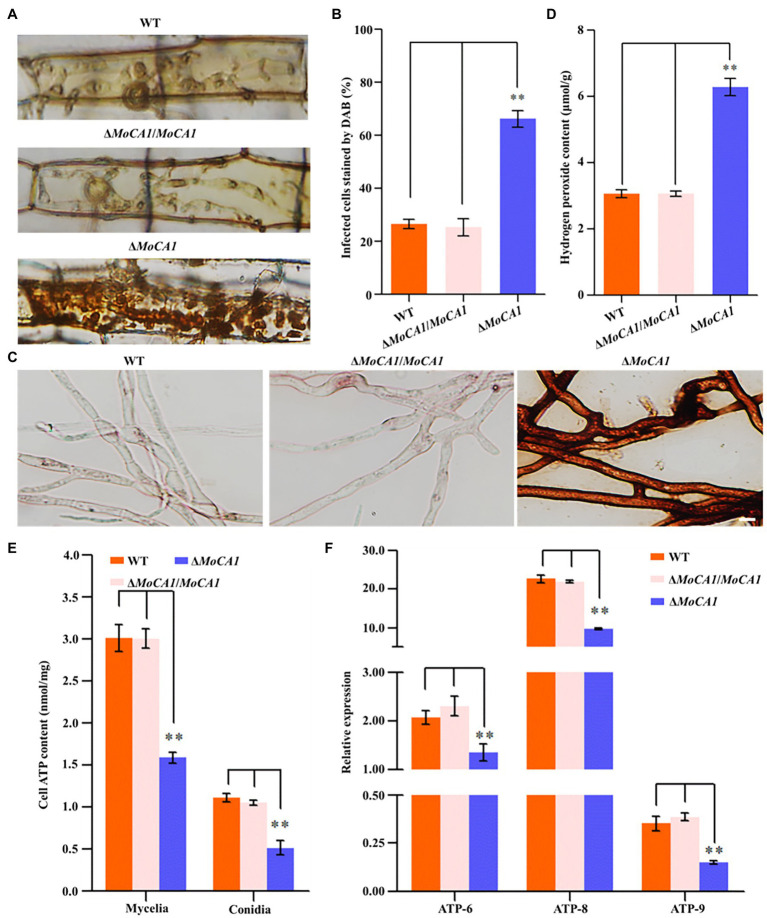
Comparison of 3,3′-diaminobenzidine (DAB) staining and endogenous H_2_O_2_ among the strains. **(A)** DAB staining of leaf sheath cells of rice infected by wild-type, mutant, and complementation strains at 36 hpi. Scale bar = 10 μm. **(B)** Statistical analysis of DAB staining of leaf sheath cells infected by different strains. **(C)** DAB staining of hyphae of the wild-type, mutant, and complementation strains. Scale bar = 10 μm. **(D)** Endogenous H_2_O_2_ assay. The strains of hyphae of endogenous H_2_O_2_ were determined as described in experimental methods. **(E)** Cellular ATP assay. **(F)** Differential ATP synthesis genes analysis on transcriptomes of the strains. The above experiments were performed in triplicate and repeated three independent times for each strain. Error bars represent the ±SD of three independently repeated samples, ^**^p < 0.01.

As rice plant accumulates more H_2_O_2_ during pathogen–rice interaction, and MoCA1 expression increases with pathogenic development of *M. oryzae*, we speculate that MoCA1 is responsible for the clearance of host-derived H_2_O_2_ during infection. So, to address the relationship between MoCA1 and endogenous H_2_O_2_, DAB staining was used to identify the endogenous ROS accumulated in *M. oryzae* infected rice leaf sheath cells at 36 hpi (Figure 5A). Leaf sheaths inoculated with the Δ*MoCA1* strains, more than 60% of the investigated infected cells were stained dark brown; in contrast, less than 25% of the infected cells were stained light brown or colorless in wild-type and Δ*MoCA1*/*MoCA1* strains (Figure 5B), displaying loss of H_2_O_2_ scavenging function in Δ*MoCA1*. Also, DAB staining was used to identify the endogenous H_2_O_2_ accumulated in the mycelia of three strains. The mycelia of Δ*MoCA1* strain were stained darker brown, displaying loss of H_2_O_2_ scavenging function in Δ*MoCA1*. Also, endogenous H_2_O_2_ was measured, two times more H_2_O_2_ was accumulated in Δ*MoCA1* than in the wild type and Δ*MoCA1*/*MoCA1* (Figures 5C,D). These results reveal that MoCA1 is responsible for regulating H_2_O_2_ levels exogenous, endogenous, or plant-derived.

Moreover, in *S. cerevisiae*, hydrogen peroxide can strongly decrease the ATP level (Osorio et al., 2003), we also tested the content of ATP in mycelia and conidia of three strains. The result showed that the content of ATP in mycelia and conidia of Δ*MoCA1* strain was lower than wild-type and Δ*MoCA1/MoCA1* strains (Figure 5E), indicating that the loss of MoCA1 affects the energy metabolism in the mutant strain, which, in turn, affects the synthesis of intracellular ATP. Furthermore, when ATP synthesis genes expression level of mtATP6 (GenBank: MGG_21007), mtATP8 (GenBank: MGG_21008), and nATP9 (GenBank: MGG_00892) was checked using qRT-PCR (Liu et al., 2020), the results showed that ATP synthesis genes were significantly downregulated in Δ*MoCA1* mutant (Figure 5F). From these results we confer, that MoCA1 is involved as a positive regulator in ATP synthesis and energy metabolism in mitochondria of *M. oryzae*.

## Discussion

Carbonic anhydrase proteins that catalyze hydration of carbon dioxide are involved in a wide range of fundamental biological processes in plants, fungi, and bacteria (Supuran, 2016; Dostál et al., 2018). However, the structural evolution has resulted in classifying these enzymes into distinct classes (Elleuche and Pöggeler, 2009; Supuran, 2018a; Dreyer et al., 2020; Sridharan et al., 2021) and the conservation of each class in different domains of life makes them a potential target for controlling biotic diseases (Nishimori et al., 2007; Innocenti et al., 2009; Syrjänen et al., 2015; Supuran, 2016; Urbański et al., 2020). Despite being well-studied in humans, higher eukaryotes, β-CAs have been analyzed in only few fungal species (Kim et al., 2020; Urbański et al., 2020; Vullo et al., 2020; Supuran and Capasso, 2021). Particularly, little is known about how these enzymes play an important role in fungal pathogenesis (Cronk et al., 2001; Dostál et al., 2018). In addition, reported fungal pathogens encoding CAs belong to animal pathogenic group, whereas no CAs in plant pathogens have been characterized yet. Although the key role of CAs in all entities is to regulate of CO_2_/HCO_3_^−^ homeostasis, but this simple looking but complex reaction plays an important role in multiple physiological and biosynthetic process (Supuran, 2018b; Vullo et al., 2020). Some studies on plant and fungal pathogens interaction have only focused on plant CAs and reported that knocking down plant CAs result in susceptibility of plant to fungal pathogens (Restrepo et al., 2005), the function of phytopathogenic fungi CAs still need to be exploited. This is the first study of functional characterization of phytopathogenic fungal CAs in which we have identified single copy of carbonic anhydrase in filamentous fungi *M. oryzae* named MoCA1, and the structural configuration of MoCA1 revealed it to be a β-class carbonic anhydrase (Figure 1D) while other putative CA genes reported previously in *M. oryzae* do not belong to Beta class of carbonic anhydrases (Elleuche and Pöggeler, 2009).

In our research, we demonstrated that MoCA1, as a β-class of carbonic anhydrase with a conserved zinc-binding core, plays its role in catalyzing the hydration of carbon dioxide. In addition to the homology in amino acid sequence with other fungal β-CAs orthologues, MoCA1 showed a close phylogenetic lineage with CAs of *S. macrospora* (Figure 1A), which have already been reported to play a crucial role in fungal development and conidial germination (Elleuche and Pöggeler, 2009). Furthermore, sequence alignment of MoCA1 amino acids with yeast and *Arabidopsis* and MoCA1 3D structure alignment showed a conserved zinc-binding core coordinated by Cys46, His102, and Cys104 (Figures 1B–D). The structures analysis of β-CAs from plants, bacteria, archaea, and *C. neoformans* have also revealed the presence of conserved two cysteine’s and one histidine residues at the active site of zinc-binding domain (Kimber, 2000; Cronk et al., 2001; Elleuche and Pöggeler, 2009). Also, MoCA1 with Zn^2+^ causes differences in the enzyme activity in comparison with no-enzyme, BSA, and MoCA1 with other metal ion (Figure 1E), implying the catalytic activity of MoCA1 in carbon dioxide hydration reaction which indeed verifies that MoCA1 has an active site containing Zn ion and conserves two Cys and one His residue for proper coordination (Nair et al., 1995; Amoroso et al., 2005; Fabre et al., 2007; Dostál et al., 2020).

Spanning from bacteria to humans, different isoforms of CAs identified are known to target different tissues and organelles (Fabre et al., 2007; Temperini et al., 2008), so it is expected that fungal CAs are also unevenly distributed within the fungal cell. In general, β-CAs localize to the cytosol, plasma membrane, mitochondrion, and chloroplast in plant (Dreyer et al., 2020). In different fungi, β-CAs are found to be localized to the cell wall, cytoplasm, and mitochondrion (Dostál et al., 2020). However, a comprehensive bioinformatics study of CAs in the filamentous fungi genomes has revealed that almost all the mycelial ascomycetes encode at least one mitochondrial plant-like β-CA isoform (Klengel et al., 2005; Innocenti et al., 2009). When fused with RFP, MoCA1 localization to mitochondrion in both hyphae and conidia (Figure 2A) was in accordance with our bioinformatics prediction analysis (Supplementary Table S1) and was similar to localization of CAs identified in some animals, plants, algae, and fungi (Nagao et al., 1994; Eriksson et al., 1996; Parisi et al., 2004; Sunderhaus et al., 2006; Fabre et al., 2007; Elleuche and Pöggeler, 2009).

As some proteins are constitutively synthesized by house-keeping genes at all developmental stages for the maintenance of primary cellular function there are some other group of proteins that are selectively expressed in response to the prevailing physiological and cellular need of the organism (Zhang and Li, 2004), for this, we observed the expression pattern of MoCA1 at different developmental stages of *M. oryzae* and found that MoCA1 expression was specifically higher at the conidiophore stalk development and infectious stage (Figures 2B,C) which shows that MoCA1 is selectively expressed during *M. oryzae* life cycle especially during conidiation and pathogenic phase. Conidiation plays a key role not only in the survival and propagation of fungi under harsh environmental conditions but also facilitates the efficient disease prolongation (Wyatt et al., 2013; Batool et al., 2021). Here we showed that the disruption of MoCA1 resulted in the impaired conidiophore formation, decreased conidiation and changes in appressorial development and melanization (Figure 3). Similarly, the deletion of CAs in *C. neoformans* and *S. macrospora* also showed similar sporulation defects (Bahn et al., 2005; Elleuche and Pöggeler, 2009). We speculate that these results may be related to the excessively accumulated CO_2_ in the MoCA1 deletion mutant thus destabilizing the acid–base homeostasis of cell as certain concentration of HCO_3_^−^ is required for efficient conidiation and meiosis (Elleuche and Pöggeler, 2009, 2010).

When pathogen enters into host system, they encounter different host immune responses, for example, production of reactive oxygen species (ROS; Torres, 2010). Extensive ROS production either in pathogen or in host result in the oxidative stress leading to cell death (Torres, 2010). The expression patterns of MoCA1 increased under oxidative stress, that is, H_2_O_2_ and NaHCO_3_ stress (Figures 2D,E) show its potential cellular defense system under oxidative stress. This property was mostly found to be a major function of carbonic anhydrase III (Lii et al., 1996), however, CA Nce103p of yeast *S. cerevisiae* also showed tolerance to oxidative stress (Götz et al., 1999). Furthermore, the accumulation of internal ROS in hyphae of MoCA1 deleted strains compared to wild type (Figures 5A,C) showed the disruption of CO_2_/HCO_3_^−^/pH sensing mechanism thus modulating the fungal aerobic metabolism that leads to the accumulation of excessive ROS in mutant.

As mitochondrion is a major organelle for ATP production in living cells (Susin et al., 1998) and during host pathogen interaction, there are indications that signaling molecules or pathways initiated by such interactions may directly or indirectly target mitochondrial components of host or pathogen resulting in the mitochondrial dysfunction, membrane potential, increased generation of mitochondrial reactive oxygen species (mROS), and cellular damage (Amirsadeghi et al., 2007). Thus, localization of carbonic anhydrase in mitochondria and its potential role in cellular defense system during oxidative stress led us to measure the ATP content in mutant strains. Compared to wild type, the content of ATP in cells and the synthesis of intracellular ATP genes in Δ*MoCA1* strain was decreased (Figures 5E,F), indicating that MoCA1 is involved in ATP synthesis and energy metabolism in *M. oryzae*. At this point, the reduced pathogenicity in Δ*MoCA1* could also be partially explained because Δ*MoCA1* was exposed to a high hydrogen peroxide stress *in vivo* and reduced intracellular ATP.

**Figure 6:**
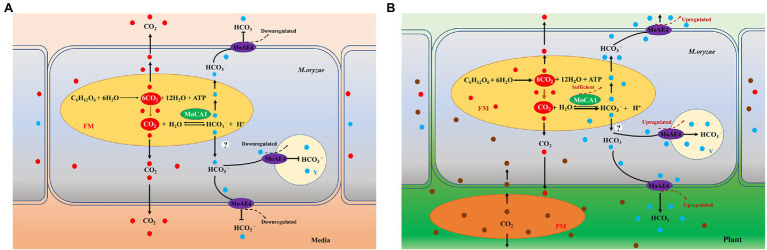
A pathogenic model mediated by MoAE4/MoCA1 system. **(A)** *Magnaporthe oryzae* is under cultivation conditions. **(B)** *Magnaporthe oryzae* infects the host cells. FM, fungal mitochondrion; PM, plant mitochondrion; V, vacuole; and MoAE4, *M. oryzae* bicarbonate transporter.

CO_2_, an important molecule, is a byproduct of mitochondrial respiration. In animals, the redundant CO_2_ needs to be released in environment, to maintain the HCO_3_^−^ + H^+^ equilibrium. The ability of HCO_3_^−^ to undergo pH-dependent conversions is central to its physiological role (Cordat and Casey, 2009). CO_2_ enters the cytoplasm through the membrane and is converted into carbonic acid (H_2_CO_3_) through spontaneous reaction. However, this acid needs to be dissociated into H^+^ and HCO_3_^−^ by intracellular carbonic anhydrases (Dang et al., 2021b). In previous research, we propose a pathogenic model mediated by MoAE4/MoCA1 system (Dang et al., 2021b), which shows that under normal growth conditions, the metabolic CO_2_ can be released freely from *M. oryzae* through spontaneous reaction and does not require the carbonic anhydrase system, and thus, both MoAE4 and MoCA1 genes are expressed at low rate because of the stability of (CO_2_ + H_2_O ⇌ HCO_3_^−^ + H^+^) reaction (Figure 6A). However, during process of invasive hyphae growth in infected plant cell, the fungal face a stressful environment which results in production of relatively high concentration of CO_2_ and low concentration of O_2_ microenvironment; and accordingly, the diffusion of fungal CO_2_ to the outside (cytosol of plant cell) is hindered. The upregulated MoCA1 will catalyze hydration of carbon dioxide to increase the concentration of HCO_3_^−^, which leads to MoAE4 upregulation for HCO_3_^−^, transportation^−^ to the vacuole or to plant cells (Figure 6B).

In this model, MoCA1 and MoAE4 are proposes to maintain the homeostasis of intracellular CO_2_^−^-HCO_3_^−^ system, which probably further ensures the intracellular acid–base balance in cells (Parks and Pouyssegur, 2015; Jeong and Hong, 2016). However, the catalysis of MoCA1 is carried out in the mitochondria, the HCO_3_^−^ transporter MoAE4 is in the cytomembrane and tonoplast. To decipher the regulation mechanism, exploring HCO_3_^−^ from mitochondria to cytoplasm, much work remains to be done.

## Data Availability Statement

The original contributions presented in the study are included in the article/Supplementary Material; further inquiries can be directed to the corresponding author.

## Author Contributions

S-HZ and YD designed the research and analyzed the data. YW, XS, and XL assisted in part of the experimental process. S-HZ, YD, and WB wrote the manuscript. All authors contributed to the article and approved the submitted version.

## Funding

This work was supported by the Local scientific research of Department of Education of Liaoning Province of China (grant no. 01032920021 to S-HZ); Special talent introduction of Shenyang Agricultural University of China (grant no. 880420019 to S-HZ); and Postdoctoral funding of Shenyang Agricultural University of China (grant no. 770221003 to YD).

## Conflict of Interest

The authors declare that the research was conducted in the absence of any commercial or financial relationships that could be construed as a potential conflict of interest.

## Publisher’s Note

All claims expressed in this article are solely those of the authors and do not necessarily represent those of their affiliated organizations, or those of the publisher, the editors and the reviewers. Any product that may be evaluated in this article, or claim that may be made by its manufacturer, is not guaranteed or endorsed by the publisher.

## Footnotes

^1^ https://www.ncbi.nlm.nih.gov/

^2^ https://zhanglab.ccmb.med.umich.edu/I-TASSER/

^3^ http://www.softberry.com/berry.phtml

^4^ http://fungalgenomics.ca/wiki/Fungal_Genomes

^5^ http://zhanglab.ccmb.med.umich.edu/I-TASSER/
