# Supplementary material for: Contribution of the Mitochondrial Carbonic Anhydrase (MoCA1) to Conidiogenesis and Pathogenesis in Magnaporthe oryzae
Source: Front Microbiol. 2022 Feb 17;13:845570. doi: 10.3389/fmicb.2022.845570 (PMC8891501; doi:10.3389/fmicb.2022.845570)

**Table S1. Identifying sub-cellular location**

| Location weights | LocDB | PotLocDB | Neural Nets | Pentamers | Integral |
| --- | --- | --- | --- | --- | --- |
| Mitochondrial | 0.0 | 0.0 | 1.56 | 1.45 | 0.00 |
| Cytoplasmic | 0.0 | 0.0 | 0.99 | 0.00 | 1.53 |
| Extracellular | 0.0 | 0.0 | 0.24 | 1.04 | 0.08 |

Note: Integral Prediction of protein location: Mitochondrial with score 8.0

**Table S2. Primers used in this study**

| **Primer name** | **Sequence 5’-3’** | **Remark** |
| --- | --- | --- |
| MoCA1-L-S | GAAGATCTTGGCAGGCAGATAACC | Amplify *MoCA1* up flank sequence |
| MoCA1-L-A | GGAATTCTTCTAACCCAGCACCC |  |
| MoCA1-R-S | GCTCTAGACGTTTCTTTCTCCCTT | Amplify *MoCA1* down flank sequence |
| MoCA1-R-A | AACTGCAGCTCCCAGATAACCACC |  |
| MoCA1-LHYG-S | AGTGGTAAATCTCCGTAAC | Verify *MoCA1* knockout mutant sequences |
| MoCA1-LHYG-A | TTTGGATGCTTGGGTA |  |
| MoCA1-RHYG-S | CTCGCCGATAGTGGAAAC | Verify *MoCA1* knockout mutant sequences |
| MoCA1-RHYG-A | TGGGAGGGCATAAACAGG |  |
| MoCA1-G-S | TACAAGGCTCACCAGAACCC | Amplify *MoCA1* sequence |
| MoCA1-G-A | GTCAAAAATGCAGCCGTGGA |  |
| MoCA1-C-S | CGCGGATCCATGGCTCAAAATCAGGATG | Amplify *MoCA1* complementation sequence |
| MoCA1-C-A | TCCCCCGGGGCGAGCCGTCATAGAG |  |
| MoCA1-PET28-S | CGCGGATCCATGGCTCAAAATCAGGATG | Protein expression primer of MoCA1 |
| MoCA1-PET28-A | ACGCGTCGACGCGAGCCGTCATAGAG |  |
| qRT-MoCA1-S | ATCCTCTGGCTTGGGTGCTCTGACT | qRT-PCR primer of *MoCA1* |
| qRT-MoCA1-A | GAGATGATGTTGGCAATGTTTCG |  |
| HYG-S | GATGTAGGAGGGCGTGGATATGTCCT | Amplify HYG sequence |
| HYG-A | AACCCGCGGTCGGCATCTACTCTATTC |  |
| Actin-S | CGTTGTTCCTATTTACGAGGG | qRT-PCR primer of *Actin* |
| Actin-A | TTGATGTCACGGACGATTTC |  |
| ATP6-S | AGTATTAGGATCAACAATCTTAGGA | qRT-PCR primer of *ATP6* |
| ATP6-A | TGCTAATCTTAACCCTAATGAGATA |  |
| ATP8-S | ATGCCTCAATTAGTTCCTTTTTA | qRT-PCR primer of *ATP8* |
| ATP8-A | CGAAAAGCTTAGATATTAATGTACG |  |
| ATP9-S | TATGGTCGAGGTCTCCAAGAA | qRT-PCR primer of *ATP9* |
| ATP9-A | CGAAACCCAGAATGGCGTA |  |

Figure S1. The construction strategies for MoCA1 protein purification, deletion and complementation strains. (A) MoCA1 expressed in *E. coli*. Purified both MoCA1 was indicated in SDS-PAGE. (B) Construction of pKD7-MoCA1:: RFP. Details of the construction are described in Materials and Methods. (C) The MoCA1 gene was replaced by the hygromycin resistant cassette (HYG). To construct the replacement vector, the flanking sequences were amplified with their corresponding primer pairs and fused with the HYG cassette. (D) Verify the target gene band of *MoCA1*. The target gene band was 766 bp. 36, 37, 40, 41 were mutants. +, wild type. -, ddH_2_O. (E) Verify the target gene band of upstream outside- HYG and HYG- downstream outside. The target gene bands were 1979 bp and 2241 bp. 36, 37, 40, 41 were mutants. -, ddH_2_O. (F) The relative expression of MoCA1 in strains. The wild type, deletion mutant, and complementation strains were detected via qPCR using cDNA as a template. The relative mRNA levels were calculated using the 2^-∆∆Ct^ method. The Actin was used as an internal standard reference. The data represent the means ± standard deviation (SD) of three experiments. The error bars represent the SD.

Figure S2. The loss of the *MoCA1* gene effect on mycelia growth, mycelial melanin, conidial germination, and appressorial development. (A) The growth of mycelia in solid medium and liquid medium. Scale bar = 1 cm. (B) Observation of conidial germination and appressorial formation. Bars = 20 μm.

Figure S3. Standard curve line. (A) Standard curve line of absorbance and hydrogen peroxide content. R^2^=0.998. (B) Standard curve line of Luminescence (RLU) and content of cellular ATP. R^2^=0.994.

**Fig S1.**


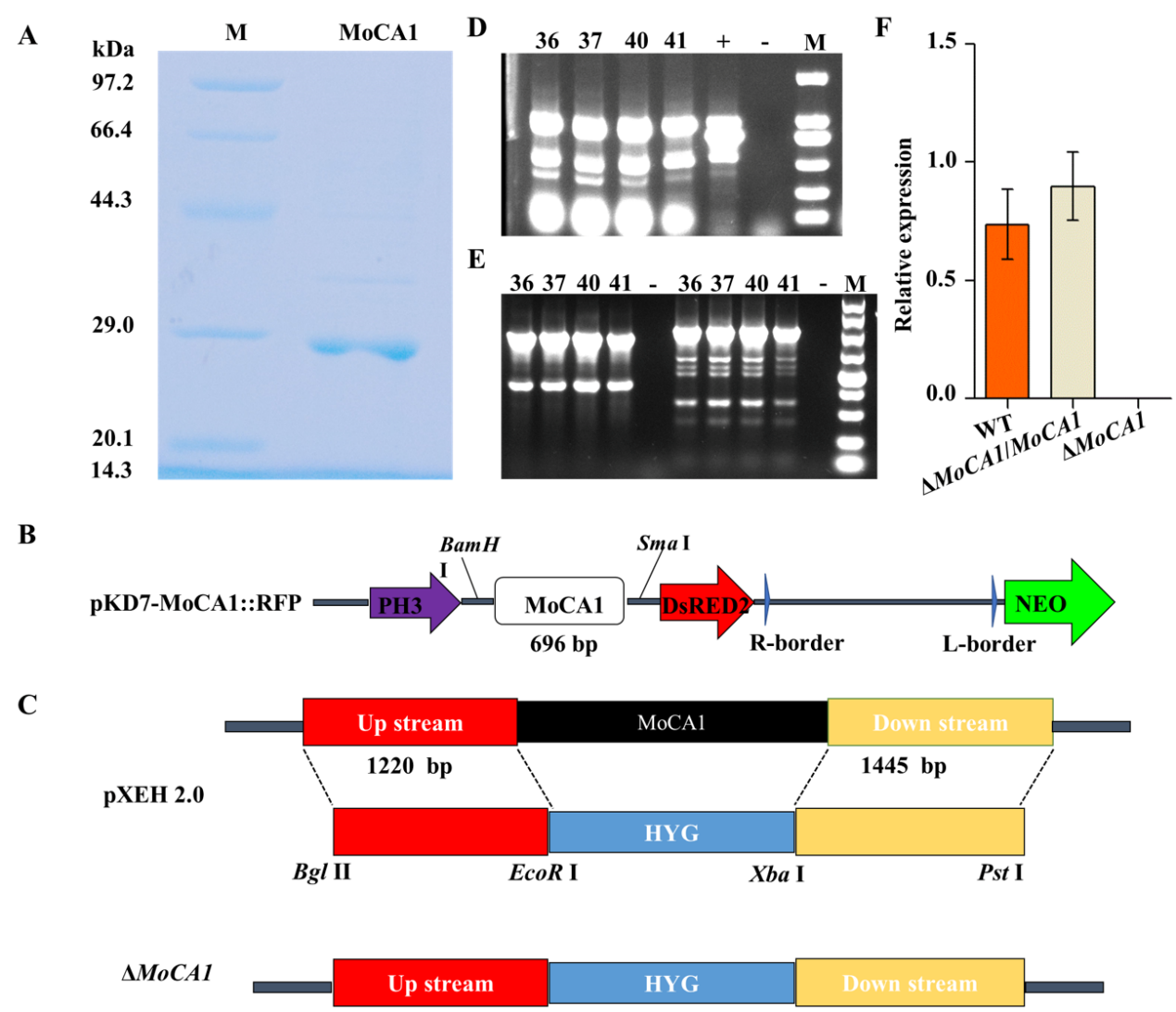


**Fig S2.**


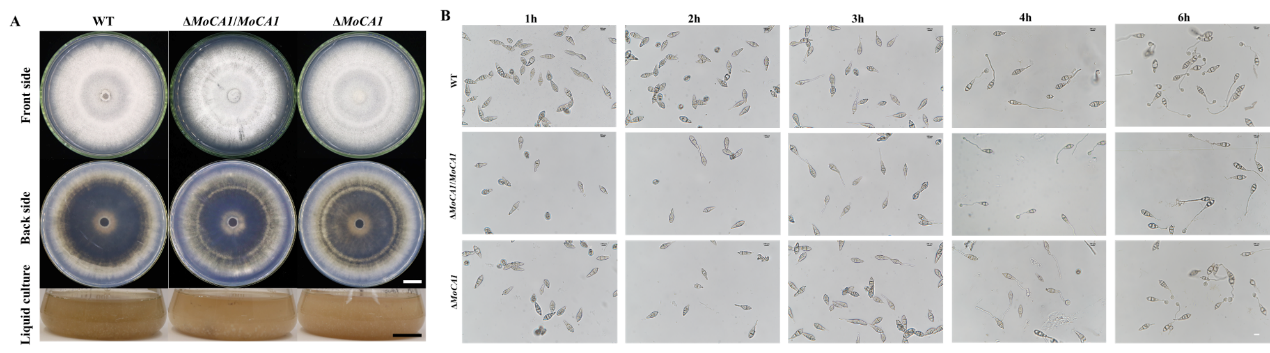


**Fig S3.**


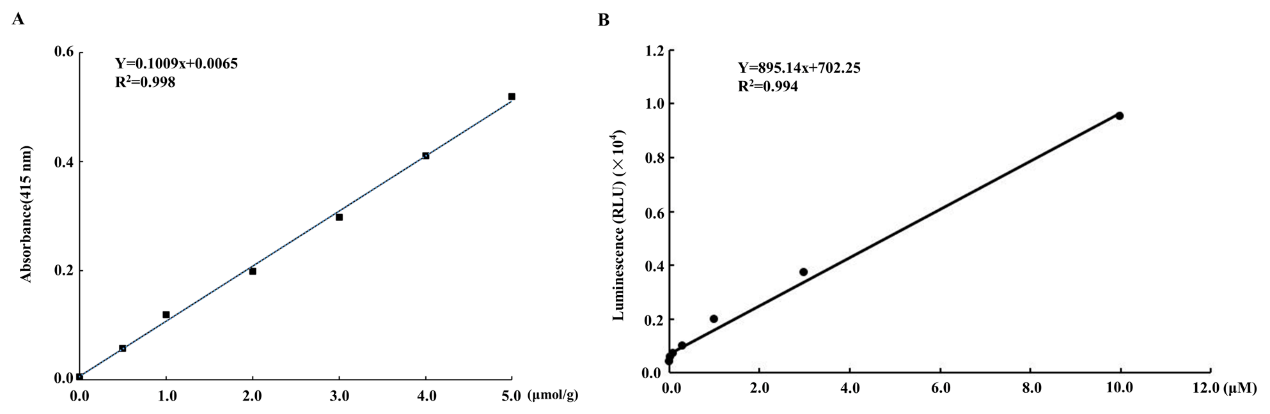

Supplement: Supplementary file 1 [file Data_Sheet_1.docx]
